# Diseases of the Ovaries

**Published:** 1873-04

**Authors:** 


### Books Reviewed.

*Diseases of the Ovaries: Their Diagnosis and Treatment.* By T. Spencer Wells. New York: D. Appleton & Co., 1873. Buffalo: Martin Taylor.

In the past few months we have had occasion to notice two excellent works upon Ovariotomy, both by American writers. The one by Dr. Peaslee stands pre-eminent as a complete treatise upon the subject. The other, like the work now before us, is mainly a history of, and observations drawn from, the clinical experience of the writer.

While American physicians have looked with pride and pleasure upon the achievements of their brethren, the publication of a work upon Ovariotomy by an author of Mr. Wells' large experience has been awaited with expectations of something which should surpass all former endeavors. It now remains to be seen if this expectation has been realized.

Mr. Wells has certainly acquired the right to speak with an authority which arises from an experienee in Ovariotomy which never has been, and perhaps, from the increasing number of operators, never will be gained by any other surgeon.

The first half of the work is about equally divided between the Anatomy and Histology of the female generative organs, together with a consideration of the pathology of the Ovaries, and the diagnosis of Ovarian tumors. The balance of the work is devoted to treatment. The author, with most English writers on surgery, tries hard to claim for British surgeons the honor of first suggesting if not practicing Ovariotomy, and goes to some length in a description of an operation in 1701, performed by an English surgeon with a lancet and splinter of fir. After laboring so hard to wrest the honor from Dr. McDowell, his tardy assertion that he was the first rational Ovariotomist is but little more of a compliment to him than the engraving on page 297, which is intended to represent Dr. McDowell.

Mr. Wells employs the bichloride of methyl as an anæsthetic which his experience has shown him is not so liable to cause vomiting as is chloroform. Of the five hundred cases reported the mortality was 25.4 per cent.; that in hospital being 26.66; in private casses 24.23.

The following is a summary of the different methods of treating the pedicle which were employed, with the results:

| | | | | |
|---|---|---|---|---|
| Clamp | 349 | cases. | 280 | recoveries. |
| Pin and ligature, acting as clamp | 15 | " | 10 | " |
| Clamp and ligature | 34 | " | 23 | " |
| Ligature returned | 57 | " | 29 | " |
| Ligature brought out | 14 | " | 6 | " |
| Cautery | 16 | " | 14 | " |
| Cautery and ligature | 14 | " | 10 | " |
| Ecrasure | 1 | " | 1 | " |
| Total | 500 | cases. | 373 | recoveries. |

No mention is made by the author of the method of Enucleation, which is, however, excusable, as at the time the book was written it had not been sufficiently brought before the profession to have reached his ears.

The whole subject of Ovariotomy is well considered, and the reports of cases drawn from his practice are interesting and instructive. The deductions to be drawn from so many cases are exceedingly interesting, and the profession is under many obligations to Mr. Wells for his careful notes.

Much of the pleasure which might arise from reading this work is destroyed by a vein of egotism and self-importance. While the profession admire Mr. Wells and his achievements, they can but censure certain self-laudations and attempts at flight in rhetoric which crop out now and then in certain portions of his work. On the whole, however, it is something to be read and studied by all interested in Ovariotomy.

